# Southeast Asia must invest in strengthening surgical systems

**DOI:** 10.7189/jogh.12.03090

**Published:** 2022-12-29

**Authors:** Ralf Martz Sulague, Xuxin Lim, Ronnie E Baticulon, Jacques Kpodonu

**Affiliations:** 1 Georgetown University School of Health, Washington, District of Columbia, USA; 2 Cebu Institute of Medicine, Cebu City, Cebu, Philippines; 3 Department of Paediatric Surgery, KKH Women’s and Children’s Hospital, Singapore; 4 Program in Global Surgery and Social Change, Harvard Medical School, Boston, Massachusetts, USA; 5 Division of Neurosurgery, Philippine General Hospital, University of the Philippines Manila, Manila, Philippines; 6 Division of Cardiac Surgery, Beth Israel Deaconess Medical Center, Boston, Massachusetts, USA

## WHY SOUTHEAST ASIA MUST INVEST IN SURGICAL SYSTEMS

In past decades, global health and development efforts in Southeast Asia (SEA) have focused on prevailing infectious diseases such as tuberculosis, malaria, and HIV/AIDS, as well as maternal and child care. While these efforts are laudable, there has been a failure to adequately address surgical diseases, which correspond to a third of the world’s disease burden [1]. These surgical diseases may range from non-communicable ones to trauma or injury and even maternal conditions that may require surgical interventions.

South and Southeast Asia have a large unmet need for surgical procedures at 5627 cases per 100 000 population, second only to Africa [2]. An estimated 3.7 billion individuals are at risk of catastrophic health expenditures (CHEs) due to surgery, with sub-Saharan Africa and Southeast Asia disproportionately affected [3]. Out of the estimated US$12.3 trillion in economic losses from 2015 to 2030 in low- and middle-income countries due to surgical conditions, US$6.1 trillion will occur in Southeast Asia, East Asia, and Oceania [2]. These unmet needs, coupled with the continuing economic losses, are strong impetus to invest in surgery.

From a systems perspective, when the capacity to perform surgery is tackled, most aspects of the complex health ecosystem are also addressed. Aside from the workforce, supply chain, and infrastructure that are also utilized in other areas of health, fundamental necessities such as electricity, water, and proper waste management are also addressed. Although this resource intensiveness makes surgical systems costly, studies have shown that investing in surgery is cost-effective due to its ability to improve care across all surgical domains and other specialities [4].

Recognition of its pivotal role in health system strengthening increased after the Lancet Commission on Global Surgery (LCoGS) and World Health Assembly Resolution WHA68.15 defined emergency and essential surgical care and anaesthesia as important components of universal health coverage. In 2021, the World Health Organization Action Framework for Western Pacific was proposed to introduce operational shifts to facilitate the provision of timely, safe, and affordable surgical care in the region, in line with achieving universal health coverage [5]. It also links with the National Surgical Obstetric and Anesthesia Plans (NSOAPs), which consist of six core domains for integrated health systems and policy development: workforce, service delivery, infrastructure, finance, governance, and information management [6].

## WHAT CAN BE DONE?

Southeast Asia is a vast region comprised of 11 nations, spanning an area of about 4.5 million square kilometres with an estimated population of 675 million people composed of diverse ethnic groups [7,8]. In this context, health care service delivery faces unique challenges, like high population density, large income inequalities, remote island distributions, and climate conditions (such as monsoons). Nevertheless, we believe several approaches can be adopted to facilitate the provision of equitable surgical care.

First, there is a need to identify current gaps in the health care systems in SEA countries through government-led top-down efforts. By taking the lead and ownership in steering the health care agenda at a national level, a tailored approach can be adopted to strengthen surgical systems by addressing health care problems specific to each country – for example, by conducting a thorough situational analysis, local needs assessment, and collecting LCoGS indicators to monitor surgical systems' effectiveness. Training and education of medical and ancillary staff should be based on those needs. Establishing health information systems is key to measuring indicators and monitoring progress to inform sound policy-making and concrete actions. National governments within the region should consider developing their own national surgical plans to synchronize country-wide efforts in addressing long-term surgical needs.

Second, the wide geographic distribution of islands in SEA poses a challenge and requires building up surgical care delivery from bottom-up. There is a need to increase surgical capacity at the district hospital level so that most of the population can access timely essential surgery, even in rural settings. First-level hospitals should be equipped to provide Bellwether procedures. Properly functioning first-level hospitals that can deliver effective basic surgical services can be one of the most cost-effective components of the regional public health system. Considering that a significant portion of health care is provided by the region’s private sector, an integrated referral network that includes the public, private, and NGO providers must be established.

Third, instead of focusing only on infectious diseases, health institutions must recognize the increasing problem of non-communicable diseases (NCD) in the region, the burden of which has dramatically increased since the 1990s and is projected to continue rising [9]. On top of these diseases, trauma or injury and maternal conditions would also need surgical interventions. The need for surgical treatment for these conditions further supports the need to strengthen surgical systems. Besides implementing preventive lifestyle measures to combat NCDs and improving prenatal screening to identify those at high risk, the government must ensure that basic surgical procedures used to treat these conditions are covered by universal health packages to reduce impoverishing out-of-pocket expenditures due to surgery.

Fourth, there is a need to move from surgical disease-focused advocacy to a more systems-oriented approach. For example, instead of separate smaller efforts each advocating for cataract, hydrocephalus, or cleft lip and palate, these advocacies can unite into a synergistic larger movement. System requirements to perform one surgical procedure are similar to another’s, just like how the requirements for caesarean section are similar to those for appendectomy [10]. Advocacy for surgical care may be directed towards strengthening local surgical systems by establishing quality infrastructure for delivering surgical care, establishing training programs in underrepresented specialities, improving financial risk protection from surgical diseases, and increasing government spending on health.

Lastly, instead of short-term surgical missions, more efforts should be put into long-term collaborations, capacity building, and sustaining local surgical ecosystems. A regional approach may be implemented through intergovernmental organizations such as the Association of Southeast Asian Nations (ASEAN), where health ministers can share their expertise and resources in implementing surgical systems to tackle common surgical problems, such as insufficient surgical workforce and essential surgical equipment procurement. For instance, the ASEAN Mutual Recognition Arrangements may be implemented and optimized to train the surgical workforce from SEA countries with few surgical training programs. Ultimately, solutions and approaches must be adapted to local contexts to optimize effectiveness.

**Figure:**
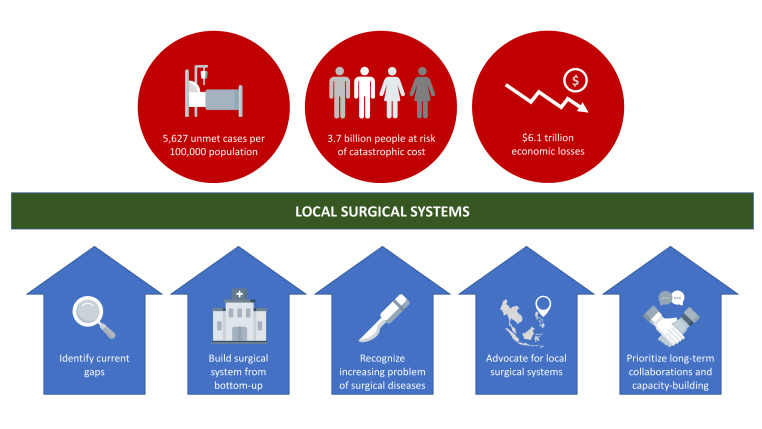
Photo: Several approaches that can be adopted to facilitate the provision of equitable surgical care in Southeast Asia. Source: created by the author (RMS).

## CALL TO ACTION

Over the last decade, global surgery has gained momentum in various regions across the globe in terms of research, advocacy, and policymaking. More ministries of health have come to realize the crucial role of surgery in public health. Southeast Asia may have a high burden of surgical diseases and a unique set of challenges to making surgery more accessible, but with the right approach and firm resolve, it can address them. Now is the time for Southeast Asia to include promoting access to safe, affordable, and timely surgical care in its health agenda to truly achieve health equity for all in this part of the world.
